# The Physiology of the Domestic Animals

**Published:** 1889-07

**Authors:** 


### REVIEW DEPARTMENT.

THE PHYSIOLOGY OF THE DOMESTIC ANIMALS. A TEXT-BOOK FOR VETERINARY AND MEDICAL STUDENTS. By ROBERT MEADE SMITH, A.M., M.D., Professor of Comparative Physiology in the University of Pennsylvania; Fellow of the College of Physicians and Academy of Natural Sciences, Philadelphia, &c. Philadelphia and London. F. A. Davis, 1889.

One would feel inclined to consider the appearance of a new text-book on general Physiology as a sort of supererogation in view of the many valuable works of the kind which have emanated in recent years from the scientific Press both of England and America, but when we consider, moreover, the rapid strides which the science of Physiology is daily making and the necessity of elaborating the data and details of such progress into some compact and systematic shape, it is evident that a good hand-book of Physiology can never prove a drug in the market. No doubt it was some feeling of this sort that compelled Dr. Smith of the University of Pennsylvania to give to the world of science the ripest fruits of his Physiological studies embodied in a thoroughly exhaustive, well written, and systematically arranged volume. Doctor Smith has not departed from the established order of Physiological writers in the sequence of his subjects, but he has introduced into each chapter the results of the most recent investigations, and has affixed to each its just scientific value. He has also wisely in our opinion, excluded all hypotheses which have not been fully established, the introduction of which have swelled Dr. Carpenter's treatises, for instance, beyond all reasonable dimensions, and which deter students from the formidable task of reading through thousands of pages. Dr. Meade's presentment of his subject is as brief as the status of the science permits, and to this much desired conciseness he has added an equally welcome clearness of statement. The work is divided into two principal parts, viz: General Physiology and Special Physiology. The first part dealing, as it does, with the law which governs all living organisms, presents no special features of significance from a comparative point of view, and is as replete with interest to the medical student as to the young veterinarian. It has however a special recommendation which cannot fail to enhance it in the eyes of the thoughtful student. Each chapter he gives with a definition or a series of definitions of the points to be considered therein, and so the attention of the reader is constantly concentrated on something which he sufficiently understands, nor is he ever compelled to retrace his steps in order to find out what he had been reading about. This is all the more important in a science which abounds with greek derivations, the elements of which are so combined as to express not only a central thought, but that thought shaded off into a great variety of relations. The author strongly insists that, as a knowledge of Physiology or of the laws that govern the normal growth and existence of organised beings is an indispensable condition for the satisfactory study of morbid processes, so an intimate knowledge of structure, or of general and minute anatomy is equally essential to an understanding of Physiology. Function is based on structure, and an adequate knowledge of the latter is the sole gateway to a proper understanding of the former. The author constantly insists on this, and indeed the student frequently finds his path barred unless his knowledge of histology is at least from fair to middling. This is another evidence of the continuity of the physical sciences and their ever growing interdependence. No one would presume now-a-days to undertake the study of biology and the development of all life without a proper knowledge of organic chemistry. We mention these facts for the benefit of those students who might undertake the perusal of Dr. Smith's treatise on Physiology without sufficient previous equipment. They should understand that the difficulties of the study are the result of their own insufficient preparation.

The veterinary student especially, unless he has been taught to regard his profession from a very elevated stand point, will not come to the study of Physiology with a broad and mature knowledge of chemistry. On the other hand the young man who possesses such knowledge, and who is anxious to keep himself abreast of the latest advances in biology will find not only instruction but a fascinating interest in Dr. Meade's chapter on cell life and cell development.

The illustrations in the work are exceedingly good and must prove a valuable aid to the full understanding of the text.

C. M. O'L.

